# Molecular Analysis of Bacterial Microbiota on Brazilian Currency Note Surfaces

**DOI:** 10.3390/ijerph121013276

**Published:** 2015-10-22

**Authors:** Tairacan Augusto Pereira da Fonseca, Rodrigo Pessôa, Sabri Saeed Sanabani

**Affiliations:** 1 Clinical Laboratory, Department of Pathology, LIM 03, Hospital das Clínicas (HC), School of Medicine, University of São Paulo, São Paulo 05403 000, Brazil; E-Mails: tairacanaugusto@hotmail.com (T.A.P.F.); farias.pessoa@unifesp.br (R.P.); 2 São Paulo Institute of Tropical Medicine, University of São Paulo, São Paulo 05403 000, Brazil

**Keywords:** bacteria, microbiome, metagenomics, currency notes

## Abstract

Currency notes have been implicated as a vehicle for transmitting community-acquired bacterial infections. However, the overall diversity of the bacterial population residing on banknotes is still unknown in Brazil. In this study, we aimed to investigate the overall bacterial population from 150 different Brazilian Rial (R$) notes in circulation using a culture-independent Illumina massively parallel sequencing approach of the 16S rRNA genes. Samples were randomly collected from three different street markets or “feiras” in the metropolitan region of São Paulo. Taxonomical composition revealed the abundance of *Proteobacteria* phyla, followed by *Firmicutes* and *Streptophyta,* with a total of 1193 bacterial families and 3310 bacterial genera. Most of these bacterial genera are of human, animal, and environmental origins. Also, our analysis revealed the presence of some potential pathogenic bacterial genera including *Salmonella*, *Staphylococcus*, and *Klebsiella*. The results demonstrate that there is a tremendous diversity of bacterial contamination on currency notes, including organisms known to be opportunistic pathogens. One of the factors that may contribute to the richness of bacterial diversity in currency notes is personal hygiene. Thus, our results underscore the need to increase public awareness of the importance of personal hygiene of money handlers who also handle food.

## 1. Introduction

Currency notes and coins are handled and circulated by individuals with different health statuses and various personal hygiene habits, and are often stored under adverse hygienic conditions. Although credit cards and electronic banking have replaced traditional money to some extent, cash exchanges are still commonly used worldwide for the purchase of goods and services. Unlike coins, paper banknotes provide a large surface area for bacterial attachment and proliferation [1] and could serve as vectors for transmission of potentially pathogenic and\or resistant microorganisms through handling [2,3]. The level of contamination and the chance of transmission of microorganisms imposed on paper currencies and coins are often associated with the economic status of a country and the levels of community hygiene [4]. The contamination could occur during production, storage, use, or handling [5,6]. High rates of microbial contamination of currency notes in circulation have been reported in multiple studies in different parts of the world [7,8,9]. For instance, 100% of currency notes from India [8,10], Bangladesh [11], Iraq [12], and Ghana [9] were found to carry pathogenic or potentially pathogenic bacteria. An early study conducted by Abrams and Waterman found that 42% of paper money collected from laboratory workers was contaminated by potential pathogens, such as *S. aureus*, *E. coli*, *Klebsiella* sp., *P. aeruginosa*, and *Proteus mirabilis* [13]. A recent study on currency paper from various countries found a high contamination of *E. coli* on banknote samples from the USA and China, and Salmonella sp. was detected only from samples in the USA, China, and Ireland, while the presence of *S. aureus* varied from country to country [1]. The same study showed that the lower the denomination of the note, the higher the typical bacterial content of the currency. The results also showed that the age of the notes and the material that was used to produce the notes correlate with the number of bacterial contaminations [1]. Lower denomination notes carry the highest number of infectious agents because they are exchanged more than higher denomination notes [14]. These results have further been supported by other studies [15,16].

Needless to say, previous studies have provided evidence that the surfaces of currency notes serve as hotspots of harmless and pathogenic bacterial contamination. However, the overall bacterial diversity in currency notes remains largely unknown, as most previous studies of money microbes relied upon cultivation-dependent techniques that preclude characterization of in-depth bacterial communities. Therefore, the aim of this study was to profile microbiota composition in circulating Brazilian paper currencies using a large-scale DNA sequencing system of the 16S rRNA gene. 

## 2. Experimental Section

A total of 150 banknotes involving five denominations (2, 5, 10, 20, and 50), 30 notes each, were randomly collected from three different street markets or “feiras” in the metropolitan region of São Paulo, the most populated city in Brazil. Apparently mutilated or damaged notes were not used in this study. Each banknote with an equal value from each feira was joined and deposited into an individual, sterile, stomacher bag as described previously [1]. All samples were frozen (−80 °C) until DNA extraction.

Prior to the extraction of DNA, the currency notes from the same denomination from the three feiras were joined and put in 50 mL sterile Falcon tubes and immersed in previously UV exposed ultra-pure water (Invitrogen, Grand Island, NY, USA). The tubes were incubated for 10 min at room temperature with vigorous agitation steps every two minutes to remove attached bacteria. After being rinsed, the bank notes were removed, and bacterial cells were harvested by centrifugation for 10 min at 9000× *g*. The supernatant was carefully discarded and the pellet was re-suspended in whatever supernatant was left. The DNA from each pellet was extracted using the PowerSoil DNA kit (MO BIO Laboratories™, Carlsbad, CA, USA) according to the manufacturer’s protocol. The V4 region of the 16S rRNA gene was amplified using the primers Bakt_341F/Bakt_805R (5′-CCTACGGGNGGCWGCAG-3′, 5′-GACTACHVGGGTATCTAATCC-3′ [17]. Amplification was performed in two steps using a custom Illumina (San Diego, CA, USA) preparation protocol where the 1st PCR was conducted with forward primers that contained partial unique barcodes and partial Illumina adapters. The remaining ends of the Illumina adapters were attached during the 2nd PCR and the barcodes were recombined *in silico* using paired-end reads. The extracted DNA from all samples was used as templates in triplicate amplification in the 1st PCR and pooled before the 2nd PCR. The 1st PCR consisted of a 25 μL solution containing 5 μL 5× HF buffer (Thermo Fisher Scientific, Waltham, MA, USA), 0.5 μL deoxyribonucleotide triphosphates (10 mM, Invitrogen, Life Technologies, Grand Island, NY, USA), 0.25 μL Phusion Hotstart II polymerase (0.5 units; Thermo Fisher Scientific Inc., Vilnius, Lithuania), 13.25 μL certified nucleic-acid free water, 0.5 μL (10 μM) forward primer, 0.5 μL (10 μM) reverse primer, and 5 μL template DNA. The 1st PCR conditions were as follows: initial denaturation for 2 min at 98 °C; 22 cycles of 20 s at 98 °C, 30 s at 50 °C and 20 s at 72 °C; and 72 °C for 2 min for final extension. After the 1st PCR, the triplicate reactions were pooled and cleaned with the Qiagen MinElute PCR Purification Kit according to the manufacturer’s protocol (Qiagen, Germantown, MD, USA). Samples were eluted in 11.5 μL Buffer EB (10 mM Tris-Cl, pH 8.5, Qiagen, Germantown, MD, USA). For the 2nd PCR, a single primer pair was used to add the remaining Illumina adapter fragments to the ends of the amplified products from the 1st PCR. The 2nd PCR was performed using the same combination of reagents that was used in the 1st PCR. The PCR cycling condition consisted of an initial denaturation step at 98 °C for 2 min, followed by 12 cycles of 98 °C for 20 s, 66 °C for 30 s and 72 °C for 20 s, and a final extension was carried out at 72 °C for 2 min. The amplified products from the 2nd PCR were separated by gel electrophoresis and purified using Freeze ‘N Squeeze DNA Gel Extraction Spin Columns (Bio-Rad, Hercules, CA, USA). Each purified amplicon was quantified on a Qubit 2.0 Fluorometer (Life Technologies, Carlsbad, CA, USA), pooled at equimolar concentration, and diluted to 4 nM. To denature the indexed DNA, 5 µL of the 4 nM library were mixed with 5 µL of 0.2 N fresh NaOH and incubated for 5 min at room temperature. Then, 990 µL of chilled Illumina HT1 buffer were added to the denatured DNA and mixed to make a 20 pM library. After this step, 360 µL of the 20 pM library was multiplexed with 6 µL of 12.5 pM denatured PhiX control to increase sequence diversity and then mixed with 234 µL of chilled HT1 buffer to make a 12 pM sequenceable library. Finally, 600 µL of the prepared library was loaded on an Illumina MiSeq clamshell style cartridge for paired end 300 sequencing. The library was clustered to a density of approximately 820K clusters/mm^2^. Image analysis, base calling, and data quality assessment were performed on the MiSeq instrument (San Diego, CA, USA). 

To confirm that the PCR reagents were not the source of bacterial sequences, PCR of the no-template control was performed. Also, prior to extraction and amplification, all reagents and ultrapure water were exposed to UV light of 254 nm for at least 3 min. No visible amplification signal was observed for the no-template control on a gel, indicating that bacterial contamination was minimal. 

The library was clustered to a density of approximately 820K clusters/mm^2^. Image analysis, base calling, and data quality assessment were initially performed on the MiSeq instrument. Any reads containing two or more ambiguous nucleotides, low quality score (average *q* score < 25), or reads shorter than 300 bp, were discarded. For the 16S primer trimming, two nucleotide mismatches to the adjacent PCR primer were allowed. MiSeq forward and reverse reads were paired using the PANDAseq v.2.9 [18] with default parameters. Potential chimera sequences were detected and removed using the UCHIME algorithm [19]. To reduce computational burden analysis, 10% of reads were randomly selected from each currency note and considered for further analysis. To avoid sampling size effects, the number of reads per sample was normalized to 1837 for each dataset by randomly subsampling to the lowest number of reads among samples. The taxonomic classification of each read was assigned against the EzTaxon-e database [20] at a 97% threshold of pairwise sequence similarity. The richness and diversity of samples was determined by Chao1 estimation and the Shannon diversity index at 3% distance. The bacterial community richness indices (non-parametric Chao1) and diversity indices (Shannon estimator) were calculated using Mothur and Shannon-ace-table.pl software programs (Chunlab Inc., Seoul, Korea). The overall phylogenetic distance between communities was estimated using the Fast UniFrac [21] and visualized using principal coordinate analysis (PCoA). To compare operational taxonomic units (OTUs) between samples, shared OTUs were obtained with the XOR analysis of CL community program v3.43 (Chunlab Inc., Seoul, Korea).

The sequencing data have been uploaded to zenodo [22].

## 3. Results and Discussion

**Figure 1:**
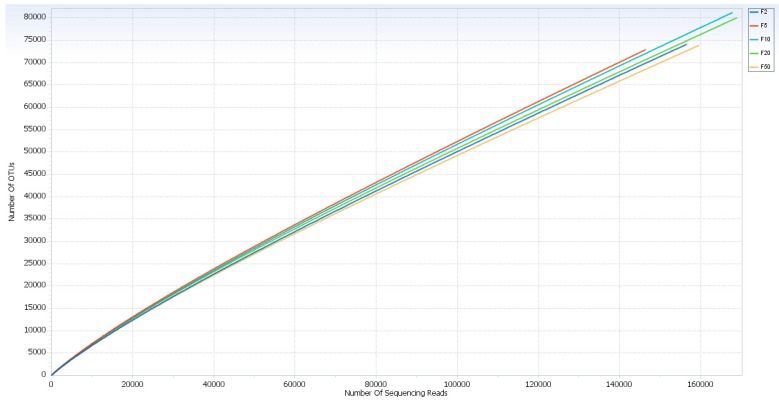
Rarefaction analysis of the five samples. Rarefaction curves of OTUs clustered at 97% sequence identity across the samples.

After extraction, the samples were found to contain DNA from 62 ng; negative controls (no cells) had no quantifiable DNA based on Qubit analysis. Using the Illumina sequencing-by-synthesis method (MiSeq platform), 10,659,379 reads in total were generated by the five samples. After filtering, 9,260,221 effective sequences remained, accounting for nearly 86.8% of the total sequences. To minimize computational time, a random 10% of reads from each sample was computationally selected. This resulted in a total of 962,982 valid reads, of which 799,460 (83%) were derived from bacterial sequences, 161,560 (16.7%) eukaryotic source, 1950 (0.2%) unmatched sequences, and 16 (0.002%) from archaea. Analyses were limited to bacterial populations. The unmatched sequences of OTUs failed to be assigned into any genus with a confidence level higher than 50%, suggesting the presence of many novel bacteria. The distribution of sequence lengths produced agreed with the amplicon length (464 bp) of the 16S rRNA. Reads ranging from 146,470 to 168,981 were contained in each of the five bacterial communities, and the OTU ranged from 72,923 to 81,215 (Table 1). The indices of bacterial diversity were estimated using a rarefaction curve (Figure 1) based on OTUs. This analysis indicated 97% similarity of OTUs at the 3% divergence was attained for each sample and suggests an adequate depth of coverage. By rarefaction analysis estimates, the trend for species richness on different currency notes was quite similar to each other. Chao1 and the Shannon index were calculated to estimate the alpha diversity. Chao1 analysis revealed a decreased trend of richness in the currency note R$ F5 and F50 compared to the currency notes R$ F2, F10 and F50. The highest richness value of Chao1 was observed in R$ F20 and the lowest in R$ F5 (Table 1). 

**Table 1:** Library reads and sequence diversity of 16 S rRNA.

Currency Note (R$)	Valid Reads	No. of OTU (>97% identity)	Chao1 Value	Shannon Index
F2	156,537	74,109	8,371,643,675	9,574,233
F5	146,470	72,923	7,528,842,108	9,858,164
F10	167,851	81,215	8,764,508,415	9,937,533
F20	168,981	80,076	8,808,417,211	9,919,012
F50	159,621	73,891	8,237,323,319	967,336

**Figure 2:**
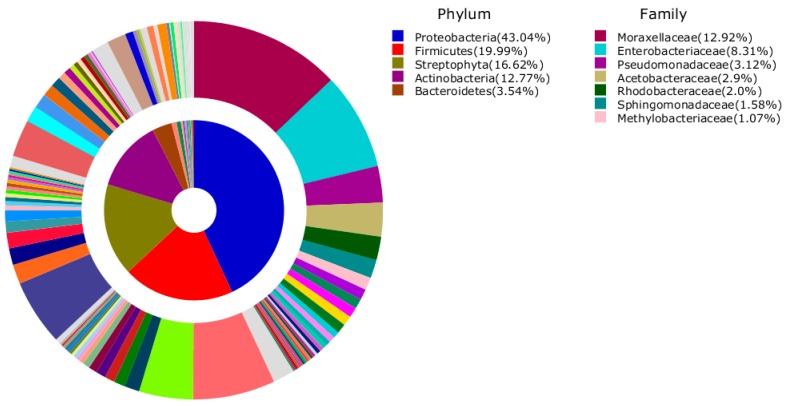
Average composition of bacteria from all samples (inner area: Phylum, outer area: Family). Phyla and Families with more than 1% of their proportion were represented.

**Figure 3:**
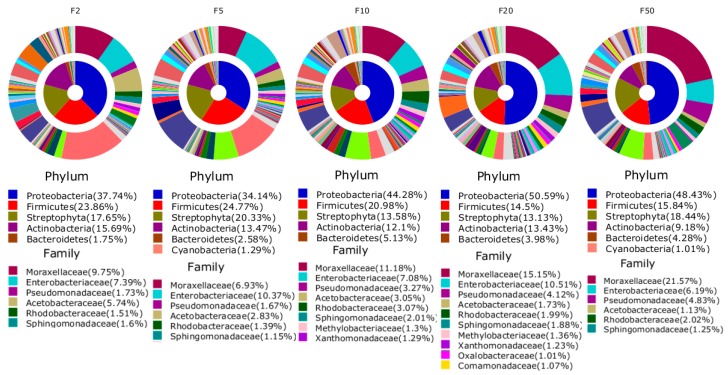
Average composition of bacteria from each sample (inner area: Phylum, outer area: Family). Only bacterial phyla and families that had a relative abundance of 1% or greater are presented.

The Shannon index computed at 3% dissimilarity showed the lowest value of evenness (9574) for the sample R$ F2 compared to the all other samples that revealed the highest value of evenness. No statistically significant difference was found in the Choa1 and the Shannon index between the groups of currency note samples. The bacteria were from 58 phyla, 198 classes, 466 orders, 1193 families, and 3310 genera. Proteobacteria (43%) was the most abundant phylum with 28.8% contributed by Gamma-proteobacteria and 17.9% by Bacilli. The most abundant OTUs at phylum and family levels that accounted for more than 1% of all sequences are shown in Figure 2. Proteobacteria were commonly most abundant in each sample accounting for 37.7%, 34.1%, 44.3%, 50.6%, and 48.4% in banknotes R$ F2, F5, F10, F20, and F50, respectively (Figure 3). The six OTUs of the most abundance species associated with the five sample libraries were related to *Staphylococcus saprophyticus* (0.4%–2.8%), *Staphylococcaceae_uc_s* (0.7%–1.4%), *Staphylococcus_uc* (0.5%–1.1%), *Moraxellaceae_uc_s* (0.6%–1.7%), *Enterobacteriaceae_uc_s* (1.1%–0.8%), and *Acinetobacter_uc* (0.5%–1.3%) (Table 2). 

**Table 2:** Identities of the six most abundant OTUs in the bacterial communities.

	F2	F5	F10	F20	F50
Abundance Order	Taxa				
	(Abundance)				
1	*Staphylococcus saprophyticus* (2.8%)	*Staphylococcus saprophyticus* (2.7%)	*Moraxellaceae_uc_s* (1.6%)	*Moraxellaceae_uc_s* (1.5%)	*Moraxellaceae_uc_s* (1.7%)
2	*Staphylococcaceae_uc_s* (1.4%)	*Enterobacteriaceae_uc_s* (1.1%)	*Acinetobacter_uc* (0.8%)	*Acinetobacter_uc* (1.3%)	*Acinetobacter_uc* (1.1%)
3	*Staphylococcus_uc* (1.1%)	*Staphylococcaceae_uc_s* (0.9%)	*Enterobacteriaceae_uc_s* (0.8%)	*Enterobacteriaceae_uc_s* (1.0%)	*Enterobacteriaceae_uc_s* (1.1%)
4	*Moraxellaceae_uc_s* (1.1%)	*Staphylococcus_uc* (0.9%)	*Staphylococcaceae_uc_s* (0.7%)	*Staphylococcaceae_uc_s* (0.7%)	*Staphylococcaceae_uc_s* (0.6%)
5	*Enterobacteriaceae_uc_s* (0.9%)	*Moraxellaceae_uc_s* (0.6%)	*Staphylococcus_uc* (0.5%)	*Staphylococcus_uc* (0.5%)	*Staphylococcus_uc* (0.6%)
6	*Acinetobacter_uc* (0.9%)	*Acinetobacter_uc* (0.5%)	*Staphylococcus saprophyticus* (0.4%)	*Staphylococcus saprophyticus* (0.5%)	*Staphylococcus saprophyticus* (0.6%)

**Figure 4:**
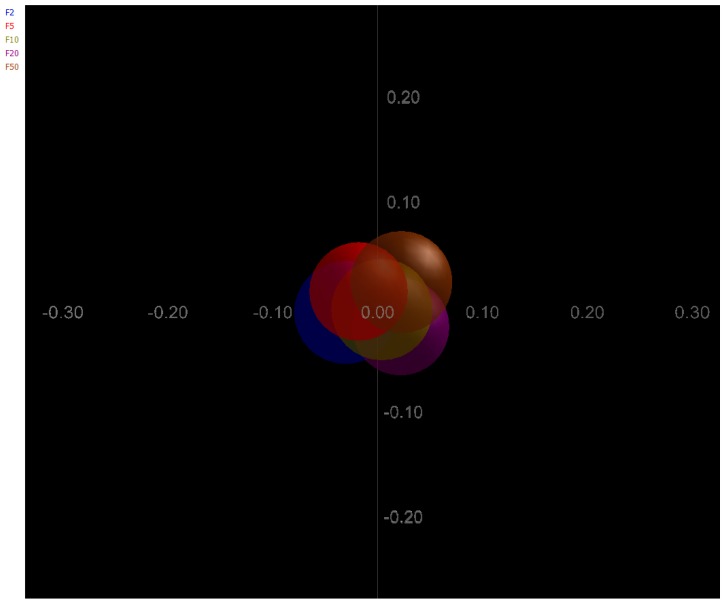
Principal Coordinates Analysis (PCoA) analysis of the microbiome of each currency note sample based upon beta div shannon 2d correlation. Different colored symbols are indicative of the various banknotes.

The weighted Principal Coordinates Analysis (PCoA) of the microbiome of each sample based upon the UniFrac method was performed to compare overall composition of bacterial community within the samples. In the two-dimensional plot visualized from the Unifrac weighted distance matrix PCoA, all samples grouped in one cluster with no apparent difference in average size of their circles (Figure 4).

The use and constant exchange of paper currency could serve as a vehicle for transmission of bacteria and other potentially pathogenic microorganisms [23]. The amount and diversity of microbes found on notes may vary depending on various factors including the general hygiene levels of the population and who is likely to be handling the money. Here, we explored the first deep sequencing analysis of microbial populations associated with currency notes collected from vegetable and fruits vendors using culture independent Illumina next generation sequencing technology. Our findings revealed that the predominant phyla (in terms of percentages and reads) were *Proteobacteria*, *Firmicutes*, *Streptophyta* and *Actinobacteria*. The prevalence of these phyla has also been reported previously in various studies using culture-dependent assays [13,23,24,25]. Within these dominant phyla, the bacterial families with the highest relative abundances across all the samples were *Moraxellaceae*, *Enterobacteriaceae*, *Pseudomonadaceae*, *and Acetobacteraceae*. From our data, it is not possible to draw any firm conclusions regarding their pathogenicity; pathogenic taxa are suspected from previous studies. Species belonging to *Pseudomonadaceae* and *Moraxellaceae* families have been reported as opportunistic pathogens such as *P. aeruginosa* [26], *M. catarrhalis* [27], and *M. osloensis* which rarely causes infections but has been associated with bacteremia and endophthalmitis [28,29]. In general, the most common bacterial genus observed in this study was found to be *Acinetobacter.* The presence of *Acinetobacter* has been frequently detected on currency notes from Egypt [25], USA [24], and Kenya [30]. In this study, we identified the presence of more than 35 *Acinetobacter* spp, (tentatively, *A. harbinensis*, *A. lwoffii group*, *A. johnsonii*, *A. tjernbergiae*, *A. pittii group*, *A. rudis*), in all currency note samples. Among them, *Acinetobacter_uc* is the most predominant bacterial species, followed by *A. harbinensis*, and *A. lwoffii* group. These species have been isolated as commensals from the skin, throat, and in secretions of healthy people and have occasionally been detected in water, soil, sewage, and in foods, arthropods, and the environment [31]. Thus, the high prevalence of *Acinetobacter* spp on these banknotes is not surprising as most of these species are part of normal human flora. It was previously thought that *Acinetobacter* spp were not a common human pathogen but during the last three decades they have emerged as infectious agents of nosocomial infections [32,33,34,35].

Community-acquired infections are less frequent but have also been reported in several studies [36,37]. Of note, Acinetobacter spp are very well adapted to survive for several weeks on abiotic surfaces under dry conditions due to their ability to form *“biofilm”* [38] and their propensity to rapidly acquire antibiotic resistance mechanisms [39,40]. On the basis of these facts, the presence of *Acinetobacter* *spp*, including *A. baumannii* in this study raise an obvious public health concern; other studies would be needed to estimate the prevalence of antibiotic resistance of these species. 

The vast majority of the bacterial species detected in this study are occasionally present in the environment and as normal flora in humans. Thus, their prevalence on currency notes is expected as they frequently come into contact with human hands. In addition, many other human-associated bacteria, including several strains associated with the mouth, urine, and intestines (e.g., *Staphylococcus saprophyticus,* *Staphylococcus aureus, Staphylococcus epidermidis,* *Klebsiella pneumoniae,* *Enterobacter faecalis*, *etc.*) were detected in our samples, which is also not surprising. The presence of *Salmonella enterica* in our study may indicate poor hygiene in the persons who handled the banknotes. Another pathogenic bacteria, *Klebsiella pneumoniae* (belonging to the bacterial family Enterobacteriaceae), associated with a wide range of infections, including cystitis, pyelonephritis, osteomyelitis, meningitis, bacteremia, septicemia, liver abscess, and wound infections [41,42,43] has been identified in our study. Similarly, *Staphylococus aureus* (belonging to the bacterial family Staphylococcaceae), which is commonly found on the skin, axillae, perineum, and in the nares of healthy individuals and can cause serious infections such as bloodstream infections, pneumonia, or bone and joint infections [44,45] was identified in this study. With all data collectively considered, identification of these bacteria may represent a public health hazard. 

Our approach to investigation reports the presence of bacterial populations regardless of whether they are dead or alive, culturable cells or non-culturable cells. Therefore, future study using RNA-based approaches, such as RNAseq, is needed to confirm the existence of viable bacterial populations on paper currencies. Despite the identification of these bacteria in our samples, it is difficult to trace the source of a community-acquired infection back to paper notes. Also, our study suggests that the Brazilian paper notes may contribute to the transmission of potentially harmful bacteria and should be a matter of concern for public health authorities.

Our analysis did not show any significant differences in bacterial population among notes with different denominations. These results are in contrast to previous studies [23,46], which demonstrated that paper currencies with lower denomination had the most contaminants compared to those with higher denominations. The differences in our results compared to other studies can partially be explained by the methodological differences among the studies. 

## 4. Conclusions 

Our study is the first of its kind to assess and provide a comprehensive assessment on the diversity in bacterial communities and presence of potential bacterial pathogens on paper currencies. As reported in the current study, a high degree of microbial diversity derived from currency banknotes may be alarmingly attributed to poor personal hygiene. Thus, these results should raise the concern of all individuals who handle paper money. To conclude, it is very important to highlight the need for proper hygienic practices for maximally reducing the spread of disease-causing pathogens. Also, microbial investigation of banknotes and replacement of contaminated notes by authorities is recommended.
